# Electronic structures of greigite (Fe_3_S_4_): A hybrid functional study and prediction for a Verwey transition

**DOI:** 10.1038/srep21637

**Published:** 2016-02-12

**Authors:** Min Wu, John S Tse, Yuanming Pan

**Affiliations:** 1 College of Materials Science and Engineering, Zhejiang University of Technology, Hangzhou, 310014, China; 2 Department of Physics and Engineering Physics, University of Saskatchewan, Saskatoon, Saskatchewan S7N 5E2 Canada; 3 Department of Geological Sciences, University of Saskatchewan, Saskatoon, Saskatchewan S7N 5E2 Canada

## Abstract

Greigite (Fe_3_S_4_) is a ferrimagnetic mineral with vital functions in both the bio-geochemical cycle and novel technological applications. However, the ground state electronic structure of this material has not been fully characterized by either experiment or theory. In the present study, *ab initio* calculations using the hybrid functional method have been performed to investigate the electronic structure and magnetic properties. It is found that the cubic structure observed under ambient temperature is a half metal and is metastable. A more stable monoclinic structure slightly distorted from the cubic form is found. The structural distortion is induced by charge ordering and associated with a metal-to-insulator transition, resulting in a semiconductive ground state with a bandgap of ~0.8 eV and a magnetic moment of 4 μB per formula unit. The results predict, similar to the magnetite (Fe_3_O_4_), a Verwey transition may exist in greigite, although it has not yet been observed experimentally.

Greigite (Fe_3_S_4_) is a ferrimagnetic mineral that was first discovered in lake sediments from California, USA^1^. It is probably formed by bacteriological reduction of iron^2^ and is widespread in nature including lacustrine sediments, magnetotactic bacterium^3^ and hydrothermal vein deposits^4^. Greigite has attracted great interests in geophysics and biology because it records the ancient geomagnetic field and environmental processes that are important for paleomagnetic and environmental magnetic studies^5^,^6^,^7^,^8^. Also, the half metallic electronic structure of greigite at ambient condition with a high curie temperature makes it a candidate for spintronic application^9^. Moreover, greigite has been suggested to be useful as an anode material in lithium-ion batteries^10^.

Similar to its iron oxide counterpart magnetite (Fe_3_O_4_)^11^, greigite has an inverse spinel structure (Fe^3+^_A_(Fe^2+^_B_Fe^3+^_B_)S^2−^_4_). Here the subscripts A and B represent the tetrahedral and octahedral iron site, respectively. Magnetite is the oldest magnetic material known to humans and has received intense investigations^12^,^13^,^14^,^15^,^16^,^17^,^18^,^19^. One of the most interesting observations is the Verwey transition, in which the resistivity abruptly increases when the temperature decreases to ~120 K^13^. This transition in the resistivity of magnetite was later found to be related to a metal-to-insulator transition induced by the charge ordering. The charge ordering at low temperature also introduces structural distortion that transforms the cubic structure at ambient condition to a monoclinic structure^20^. In view of the similarity in the crystal structure of both greigite and magnetite, Verwey transition is likely to occur in greigite^21^. However, so far no direct confirmation has been reported. A local coercivity minimum was observed in greigite at 130 K, which is close to the Verwey transition temperature of magnetite^21^. However, the local coercivity minimum was suggested to be associated with domain walls present in the samples. In another study^10^, electrical measurement showed the microcrystalline Fe_3_S_4_ was metallic at temperature as low as 5 K, indicating no Verwey transition.

In comparison with magnetite greigite is less studied. This is not only due to a much later discovery of this mineral but also due to the challenges in obtaining high-quality samples for experimental measurements. In particular, greigite is thermodynamically unstable relative to pyrite (FeS_2_)^22^. Therefore, in natural environments, greigite commonly occurs in fine-grained mixtures with pyrite. A number of methods have also been reported to synthesize greigite^23^,^24^,^25^,^26^,^27^,^28^,^29^. But, it is still very difficult to synthesize greigite samples with high purity and high crystallinity. The purity and crystallinity of greigite samples can severely affect the results of the magnetic properties and other measurements. For instance, the initial magnetic moment per formula unit of greigite was reported to be ~2.2 μB^2^,^29^ but was increased dramatically to ~3.7 μB with an improved sample^10^. The latter value, however, is still lower than that of magnetite of ~4.0 μB per formula unit^30^. It is believed that the quality of greigite samples is responsible for the discrepancy and the apparent absence of Verwey transition.

Despite previous theoretical studies, the ground state electronic structure and magnetic property of greigite remain unclear and need to be further investigated. An *ab initio* calculation using the generalized gradient approximation (GGA) augmented with an on-site Hubbard U_eff_ parameter (GGA + U) on the iron atom had been performed^29^. From matching the calculated magnetic moment to the available experimental values, it was suggested that a U_eff_ value of 1 eV would give an accurate description of the properties of greigite with a half metallic ground state. However, the calculations showed the results are highly sensitive to the choice of the U_eff_ value. As well, the dependence of the U_eff_ value on the environment and oxide state of the iron atom was not considered. Different U_eff_ values have been proposed for Fe 3*d* orbitals in different materials (*e.g*. a much larger U_eff_ value of 3.2 eV was used for magnetite^31^). The U_eff_ value is also dependent on the exchange correlation functional used^32^. Moreover, the initial spin state was not set to be the inverse spinel structure in the GGA + U calculation^29^ (*i.e.* mixed valence Fe^2+^Fe^3+^ was not considered at the octahedral sites). Therefore no charge ordering induced distortion was observed and the monoclinic structure similar to Fe_3_O_4_ was predicted to be energetically unfavorable. To overcome the empiricism of the Hubbard+U model, hybrid functionals that mix a portion of the exact nonlocal exchange of Hartree–Fock theory with the exchange density functional are often used to provide a “parameter free” and more reliable description of the electronic structure. In the present study, *ab initio* calculations using the Heyd-Scuseria-Ernzerhof screened hybrid functional (HSE06)^33^ were performed to investigate the ground state electronic structure and magnetic properties of greigite. The results are found to be sensitive to the initial choice of the spin configuration. For example, the greigite structure remains cubic when the spin configuration was initialized with all the octahedral Fe sites having the same spin. In this case, the ground state is half-metallic and is consistent with the previous study^29^. However, when the calculation was initialized with the inverse spinel spin state (Fe^3+^_A_(Fe^2+^_B_Fe^3+^_B_)S^2−^_4_), charge ordering induced distortion is found and leads to an energetically more stable monoclinic structure. The monoclinic structure is a semiconductor with a net magnetic moment value of 4.0 μB per Fe_3_S_4_ formula unit. The presented results suggest that, similar to magnetite, a Verwey transition can occur in greigite at low temperature. The theoretical results encourage further measurements of greigite with a high purity and high crystallinity sample in order to verify this prediction. In magnetite, the Fe:O stoichiometry is known to have profound effects on the observed Verwey transition^13^ and the measured magnetic moment per formula unit also vary with the particle sizes and surface structures^34^,^35^.

## Results and Discussion

**Figure 1:**
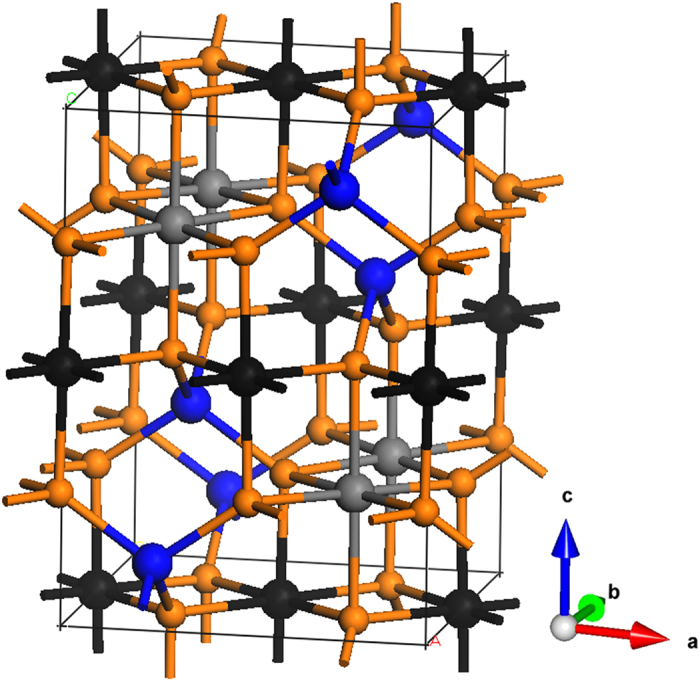
The 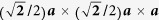 model of Fe_3_S_4_ containing 28 atoms. The brown spheres denote the sulfur atoms and the rest are three types of Fe atoms. The blue spheres are Fe atoms at the tetrahedral sites, the black and the grey spheres are Fe with different oxidation states at the octahedral sites.

In the present study, a 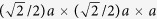 model constructed from the conventional cubic unit cell of greigite with *a* = 9.876 Å^1^ and consisted of 28 atoms (4 formula units) was used for the HSE calculations (Fig. 1). The Anderson condition that requires two Fe^3+^ and two Fe^2+^ ions in each corner-sharing Fe_B_ tetrahedron was used in the charge ordering calculation^36^. HSE calculation on a more complicated charge ordering scheme will require a much larger model and is beyond our available computational resource. Two different initial spin states were studied. The first model assumed a magnetic moment of 4.0 μB on each Fe at the tetrahedral sites, and −4.0 μB on each of the octahedral sites (HSE(I)). In the second model (HSE(II)), the inverse spinel configuration with charge ordering is considered. In the HSE(II) model, the initial spin was set to 4.0 μB at the tetrahedral Fe sites, −4.0 μB at half of the octahedral sites and −3.0 μB on the remaining half of the octahedral sites. To verify the validity of the HSE method on iron containing compounds, test calculations on magnetite were performed. An insulating ground state with the inverse spinel monoclinic magnetite structure with a net magnetic moment of 4.0 μB per Fe_3_O_4_ formula unit was obtained (See supplementary information, Fig. S1). The result is consistent with a previous study^18^ indicating that the HSE method is appropriate for greigite.

### PBE calculations

The structural information and magnetic moment of Fe_3_S_4_ computed with different methods are summarized and compared in Table 1. Calculations using the PBE functional predicted a low spin (1.9 μB per Fe_3_S_4_ formula unit) cubic structure with a lattice parameter of 9.467 Å. The lattice parameter is 4.1% shorter than the experimental value of 9.876 Å^1^. The magnetic moment is also much smaller than the experimental value of 3.7 μB^10^. These results show the localized nature of Fe 3*d* orbitals is not correctly described by the PBE functional, leading to an itinerant ground state. The itinerant Fe 3*d* electrons participate in the bonding and overestimate the interatomic interactions leading to a smaller volume and a low spin state. The density of state (DOS) and projected DOS (pDOS) analyses show that the electronic structure from the PBE functional is metallic with Fe 3*d* orbitals dominating the Fermi level (Fig. S2).

### Cubic structure from the HSE(I) calculation

**Figure 2:**
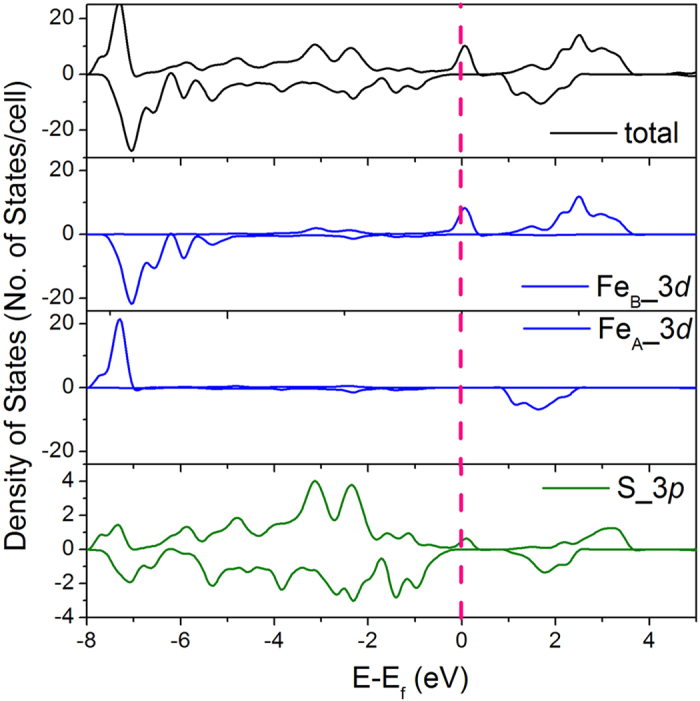
DOS and pDOS of Fe_3_S_4_ optimized cubic structure from the HSE(I) calculation. The black line is the total DOS. The blue lines are the pDOS of Fe 3*d* orbitals. The green line is the pDOS of S 3*p* orbitals. The Fermi level is adjusted to 0 eV and marked as the red dashed line.

As mentioned above, hybrid functional methods such as the HSE may be more appropriate for highly correlated systems. The optimized structure from the spin model I (HSE(I)) assuming a ferrimagnetic arrangement of the spins at the tetrahedral and octahedral sites remains to be cubic due to the absence of charge ordering. The calculated cubic lattice parameter *a* of 10.029 Å is only 1.5% larger than the experimental value. The result shows the hybrid functional HSE method indeed performs much better than the PBE functional discussed above. Improvements are also evident in the interatomic distances: The Fe_A_-S and Fe_B_-S distances are 2.257 and 2.459 Å, which are substantially longer than the PBE values of 2.133 and 2.320 Å. The net magnetic moment per Fe_3_S_4_ formula has increased to a more reasonable value of 4.0 μB. The optimized cubic structure is a half metal that is conductive in one spin channel but insulating in the other. The result agrees with the experimental observation at ambient temperature. It is noteworthy that calculations employing the PBE functional produced a metallic state in contradiction to the experiment. As shown in Fig. 2, the spin-up Fe_A_ 3*d* orbitals and the spin-down Fe_B_ 3*d* orbitals are completely filled and locating about 1.0 eV below the Fermi level. The spin polarized electrons at the Fermi level are dominated by Fe_B_ 3*d* orbitals with minor contributions from the spin-polarized S 3*p* orbitals. It is interesting to note that a bandgap is opened in both spin channels of the tetrahedral Fe_A_ atoms, indicating that the spin current can only travel through the channel connected by the octahedral Fe_B_ atoms and the sulfur atoms. The pDOS of all octahedral Fe_B_ atoms are identical, showing that there is no charge ordering. The absence of any charge ordering can be understood as follows: the valence electrons are hopping between the octahedral Fe atoms, resulting in an identical valence state of the octahedral Fe_B_ atoms rather than the expected inverse spinel state of (Fe^3+^)_A_(Fe^2+^_B_Fe^3+^_B_)S^2−^_4_.

### Monoclinic structure from the HSE(II) calculation

In the HSE(I) calculation above, no charge ordering is considered in the initial spin state. Although the calculated electronic structure is consistent with the experimental result at ambient temperature, it may be a metastable state. In contrast, the HSE(II) geometry optimization calculation that initialized with a charge ordered state, resulted in a monoclinic structure slightly distorted from the cubic precursor with lattice parameters *a* = 7.120 Å, *b* = 7.090 Å, *c* = 10.080 Å and β = 90.010° (Table 1). Most significantly, the monoclinic structure is energetically more favorable with a total energy difference of 0.377 eV per Fe_3_S_4_ formula unit lower than the cubic structure from the HSE(I) calculation. The cubic-monoclinic structural distortion in greigite is very subtle. The calculated X-ray diffraction (XRD) pattern of the monoclinic structure is almost identical to the cubic structure (Fig. S3) indicating it is very difficult to distinguish the two structures by XRD measurements. To verify that the small distortion is not due to numerical errors, we repeated the geometry optimization in two steps. First only the internal coordinates were optimized with a fixed cell, then followed by a full optimization of both the cell and the atomic coordinates. This two-step procedure produced the same monoclinic structure.

**Figure 3:**
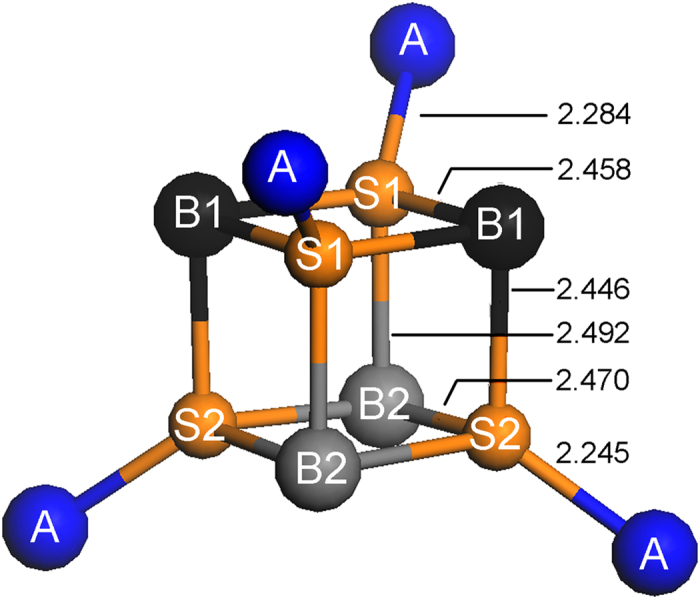
Illustration of the local structure of the optimized monoclinic structure from the HSE(II) calculation. S represents the sulfur atom, A represents the tetrahedral Fe atom, and B1 and B2 represent the octahedral Fe^3+^ and Fe^2+^ atoms, respectively. The Fe-S bond distances are in Å.

In the monoclinic structure, the net magnetic moment is 4.0 μB per Fe_3_S_4_ formula unit. As a result of the distortion, there are now several different Fe-S bonds. (Fig. 3) Unlike the HSE(I) calculation in which all the octahedral Fe_B_ atoms are identical, Bader atomic charge analysis shows that the octahedral Fe_B_ atoms in the HSE(II) calculation separate in two distinctive groups, confirming that a charge ordering state is obtained. From Bader charge analysis^37^, the Fe_B1_ and Fe_B2_ atoms illustrated in Fig. 3 have an average charge of 6.70*e* and 6.89*e*, respectively. Each Fe_B1_ atom donated 1.30*e* to their nearest neighbor sulfur atoms, slightly more than the 1.11*e* from the Fe_B2_ atoms. Thus, the Fe_B1_ atoms should be formally in the Fe^3+^ state, a higher oxidation state than the Fe^2+^ of the Fe_B2_. The charge difference between the Fe_B1_ and Fe_B2_ atoms is much less than the formal charge difference of one electron between Fe^3+^ and Fe^2+^ ions. This is consistent with the result of the previous study on magnetite^18^. The contrasting Fe-S bond lengths can be explained by the different oxidation states of the Fe atoms. For this purpose, it is easier to focus on the local structure of the sulfur atoms since they are all 4 coordinated. Due to the charge ordering (*i.e.* different oxidation state on the octahedral Fe atoms), the sulfur atoms can also be separated into two groups ((2Fe_B1_-Fe_B2_-Fe_A_)S_1_ and (Fe_B1_-2Fe_B2_-Fe_A_)S_2_). The tetrahedral Fe_A_ atoms have a smaller coordination number than the octahedral Fe_B_ atoms, so the charge donation of the Fe_A_ atom should be larger than the Fe_B_ atoms. Thus, the order of the charge transfer of the Fe-S bonds should be Fe_A_-S > Fe_B1_-S > Fe_B2_-S. On the other hand, charge transfer in Fe_B2_-S_2_ should be larger than Fe_B2_-S_1_, because the S_1_ atom already obtained more electrons from the nearest neighbor (2Fe_B1_-Fe_A_) than the S_2_ atom that has a nearest neighbor of Fe_B1_-Fe_B2_-Fe_A_. From the charge transfer analysis, the trend in the bond strength is expected to be Fe_A_-S_2_ > Fe_A_-S_1_ > Fe_B1_-S_2_ > Fe_B1_-S_1_ > Fe_B2_-S_2_ > Fe_B2_-S_1_. This analysis is consistent with the order of the bond length predicted from the HSE(II) calculation.

**Figure 4:**
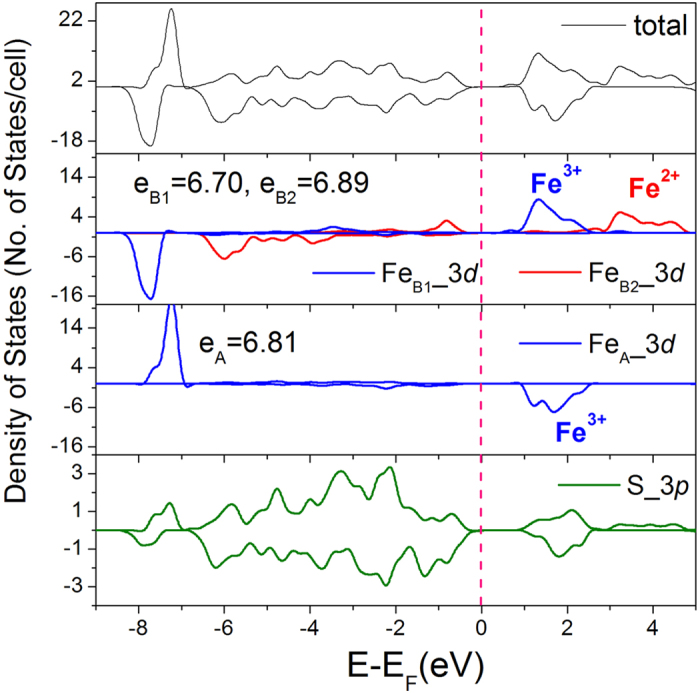
DOS and pDOS of the Fe_3_S_4_ optimized monoclinic structure from the HSE(II) calculation. e_B1,_ e_B2_ and e_A_ are the Bader charges on Fe_B1_, Fe_B2_ and Fe_A_ sites, respectively.

The electronic band structure and density of states (DOS) are shown in Fig. S4 and Fig. 4, respectively. The charge ordering induced structural distortion is accompanied by a metal-to-insulator transition resulting in a ferrimagnetic semiconductor with a bandgap of ~0.8 eV. Consistent with the atomic charge analysis, the pDOS also shows two groups of octahedral Fe_B_ atoms. Compare with the Fe_B2_ atom, the Fe_B1_ atom has a larger number of charges transferred to the sulfur atoms and the orbital energies become lower as the valence electrons are less screened. Above the Fermi level, both of the Fe_B1_ and Fe_B2_ atoms have a peak in the conduction band separated by ~2 eV. The peak of the higher oxidation Fe_B1_ is located at ~2 eV above the Fermi level, the same as that of the tetrahedral Fe_A_ atom. It is significant to point out that, the charge ordering is accompanied by a concomitant spin ordering. The Fe_B2_^2+^ ion has an integrated magnetic moment of 3.46 μB/atom, which is smaller than the Fe_B1_^3+^ ion (3.87 μB/atom) due to the extra spin-up electrons. The pDOS results support the conclusion that the ground state of the monoclinic Fe_3_S_4_ structure is an inverse spinel state of Fe^3+^_A_(Fe^2+^_B_Fe^3+^_B_)S^2−^_4_. The net magnetic moment of 4.0 μB per Fe_3_S_4_ formula unit derived mainly from the Fe atoms with minor contributions from the sulfur atoms.

To further examine the validity of the HSE results, GGA+U calculations using different U values were performed. Similar results with the HSE calculations were obtained. GGA+U calculations starting with the initial spin as HSE(I) and HSE(II) also resulted in cubic structures and monoclinic structures, respectively. Moreover, from the comparison of the total energies shown in Fig. S5, the monoclinic structure is found to be more stable than the cubic structure when the U value is larger than 1 eV. Therefore, results obtained from the *ad hoc* correction of GGA with an empirical Hubbard U on the Fe atoms are consistent with the HSE calculations presented above, thus validating our conclusions.

## Conclusions

The electronic structure of greigite (Fe_3_S_4_) has been studied by the *ab initio* hybrid functional HSE method. A metastable half-metallic cubic structure is obtained when the calculation was initiated without the consideration of charge ordering. This result is consistent with experiments at ambient temperature suggesting that greigite is a potential spintronic material. In contrast, when charge ordering is considered, the inverse spinel state Fe^3+^_A_(Fe^2+^_B_Fe^3+^_B_)S^2−^_4_ becomes most stable. The converged charge ordering state has a distorted monoclinic structure, and is more stable than the cubic structure by 0.377 eV per Fe_3_S_4_ formula unit. The net magnetic moment of the monoclinic structure is 4.0 μB per Fe_3_S_4_ formula unit, which is similar to that of magnetite. The spin polarization was derived mainly from the Fe atoms with minor contributions from the sulfur atoms. The structural distortion is associated with a metal-to-insulator transition and the monoclinic structure becomes a ferrimagnetic semiconductor with a bandgap of ~0.8 eV. Due to the high computational demand of HSE calculations, the trimer on order proposed for magnetite^19^ will require a much larger structural model and is beyond our computational ability. The present results predict the Verwey transition, which has not yet been observed experimentally, may exist in greigite. Further experiment with high purity and high crystallinity samples is needed to confirm this prediction.

## Method

Total energy and electronic structure calculations presented in this study were performed using the Vienna Ab initio Simulation Package (VASP)^38^. The projector augmented wave (PAW) method^39^ was used to describe the valence configuration, 3d^6^4s^2^ for Fe and 3s^2^3p^4^ for sulfur. The generalized gradient Perdew, Burke, and Ernzerhof (PBE)^40^ exchange-correlation density function was employed. Plane-wave expansion with an energy cutoff of 400 eV was used. The Brillouin zone is sampled with a 6 × 6 × 4 Monkhorst–Pack *k*-point grid. All calculations were performed in the spin-unrestricted method without spin-orbit coupling. Convergence in geometry optimization was reached when the Hellmann-Feynman forces on the internal coordinates and the cell parameters are better than 0.006 eV/Å. The energy convergence criterion for the electronic self-consistent calculation was 10^−4 ^eV.

## Additional Information

**How to cite this article**: Wu, M. *et al.* Electronic structures of greigite (Fe_3_S_4_): A hybrid functional study and prediction for a Verwey transition. *Sci. Rep.* **6**, 21637; doi: 10.1038/srep21637 (2016).

## Supplementary Material

Supplementary Information

## Figures and Tables

**Table 1:** Structural information and magnetic moment of Fe_3_S_4_ from different calculations.

	PBE	HSE (I)	HSE (II)
a	6.695(9.468)	7.091(10.029)	7.120
b	6.695(9.468)	7.091(10.029)	7.090
c	9.468	10.029	10.080
β	90.000	90.000	90.009
magnetic moment	1.9	4.0	4.0
Fe_A_-S	2.133	2.257	2.245 (Fe_A_-S_2_)
			2.284 (Fe_A_-S_1_)
Fe_B_-S	2.320	2.459	2.446 (Fe_B1_-S_2_)
			2.458 (Fe_B1_-S_1_)
			2.470 (Fe_B2_-S_2_)
			2.492 (Fe_B2_-S1)

HSE(I) and HSE(II) are calculations with different initial spin states. (see text) The numbers in the brackets are lattice parameters corresponding to the cubic cell. The units of distance and magnetic moment are in Å and μB, respectively.
