# Supplementary material for: Electronic structures of greigite (Fe3S4): A hybrid functional study and prediction for a Verwey transition
Source: Sci Rep. 2016 Feb 12;6:21637. doi: 10.1038/srep21637 (PMC4751502; doi:10.1038/srep21637)
Supplement: Supplementary Information [file srep21637-s1.pdf]

Supporting information

Electronic structures of greigite (Fe_3S_4): A hybrid functional study and prediction for a Verwey transition

Min Wu[1,2,3,*], John S Tse[2,*] and Yuanming Pan[3]

[1]College of Materials Science and Engineering, Zhejiang University of Technology, Hangzhou, 310014, China

[2]Department of Physics and Engineering Physics, University of Saskatchewan, Saskatoon, Saskatchewan S7N 5E2 Canada

[3]Department of Geological Sciences, University of Saskatchewan, Saskatoon, Saskatchewan S7N 5E2 Canada

Corresponding author: john.tse@usask.ca & wumindt2@163.com

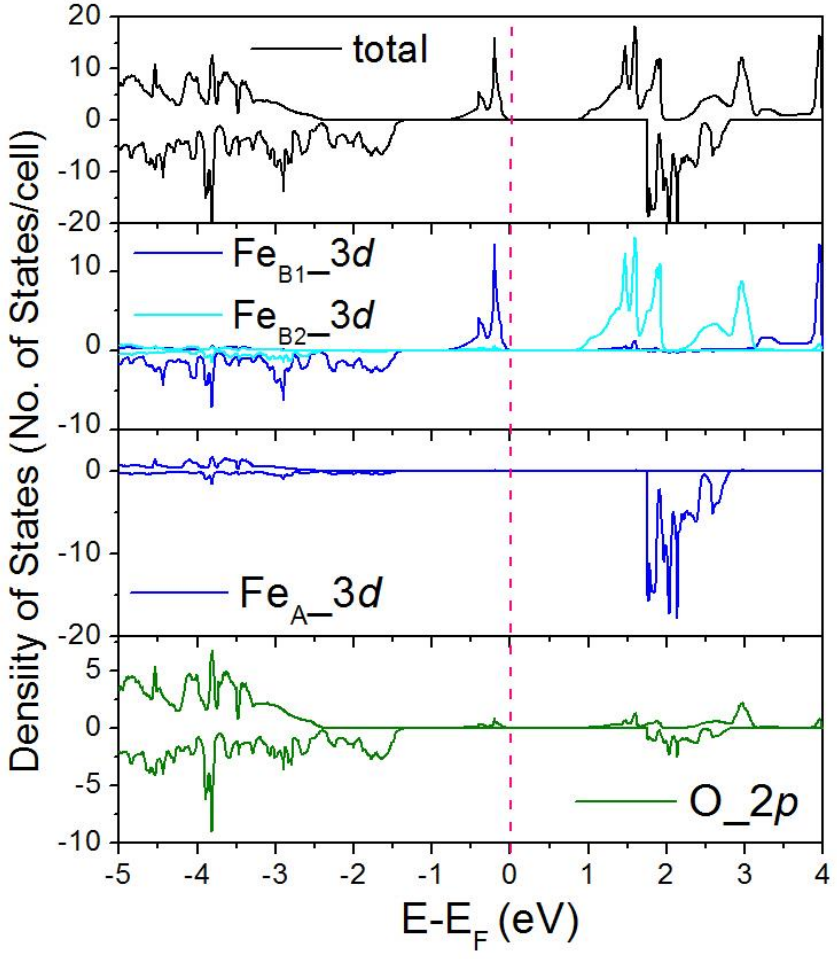

Fig. S1 The DOS and PDOS of monoclinic Fe_3O_4 structure from HSE calculation. The black line is the total DOS. The blue and light blue lines in the second column are the pDOS of octahedral Fe_{B1} and Fe_{B2} 3*d* orbitals, respectively. The blue line in the third column is the pDOS of tetrahedral Fe_A 3*d* orbitals. The green line is the O 2*p* orbitals. The Fermi level is adjusted to 0 eV and marked as red dashed line.

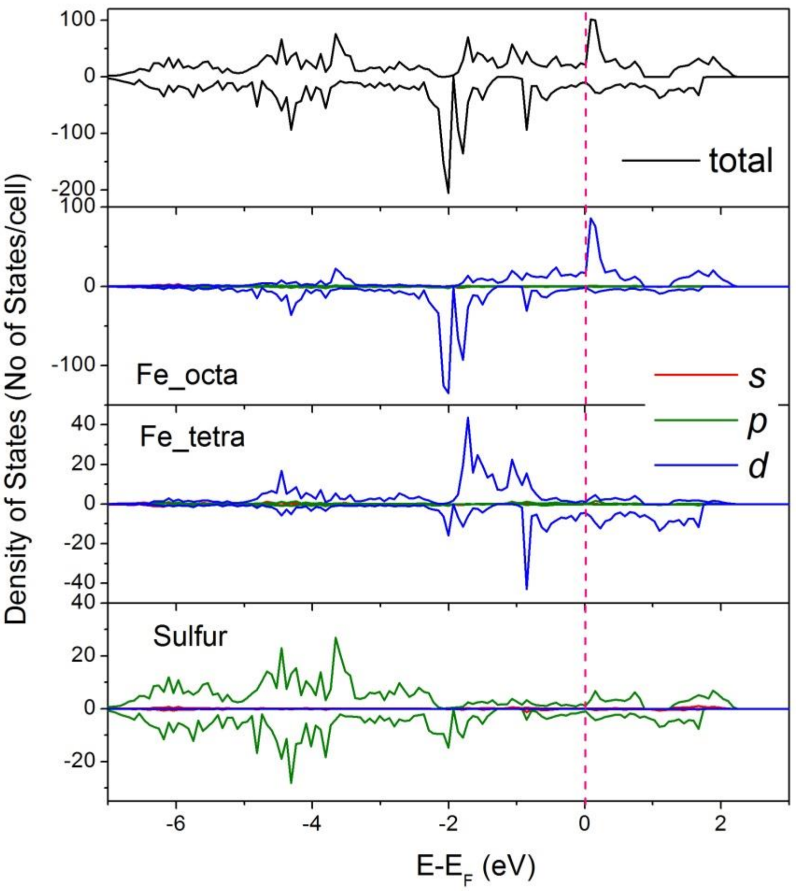

Fig. S2 The DOS and PDOS of cubic Fe_3S_4 (*Fd-3m*) structure from PBE calculation. The black line is the total DOS. The red lines are pDOS of *s* orbitals. The green lines are pDOS of *p* orbitals. The blue lines are pDOS of *d* orbitals. Fe_octa and Fe_tetra are the octahedral Fe atom and tetrahedral Fe atom, respectively. The Fermi level is adjusted to 0 eV and marked as red dashed line.

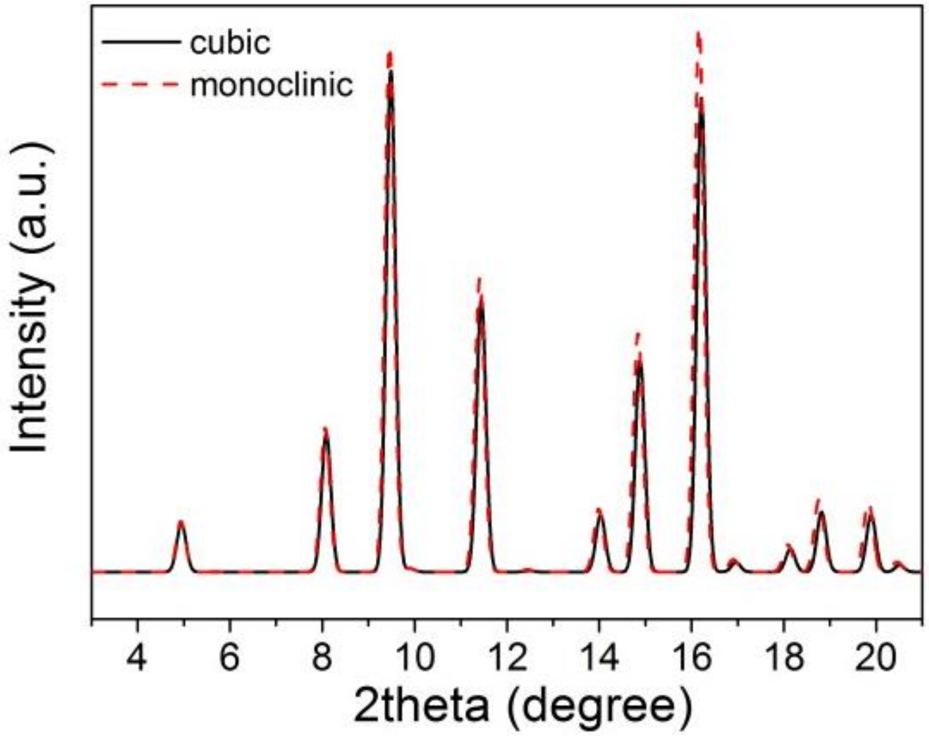

Fig. S3 Calculated powder X-ray diffraction (XRD) patterns of monoclinic (red dashed line) and cubic (black solid lines) structures of greigite (wave length λ= 1.5 Å).

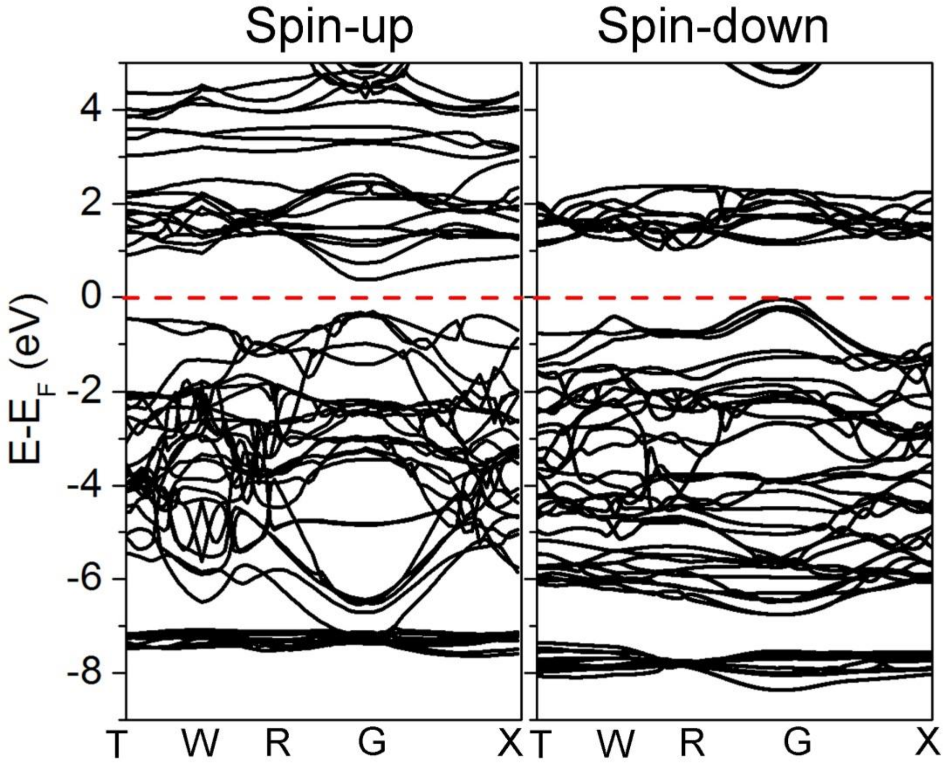

Fig. S4 Spin polarized electronic band structure of the monoclinic Fe_3S_4 structure. The left column is spin-up band structure and the right column is spin-down band structure. The Fermi level is adjusted to 0 eV and marked as red dashed lines.

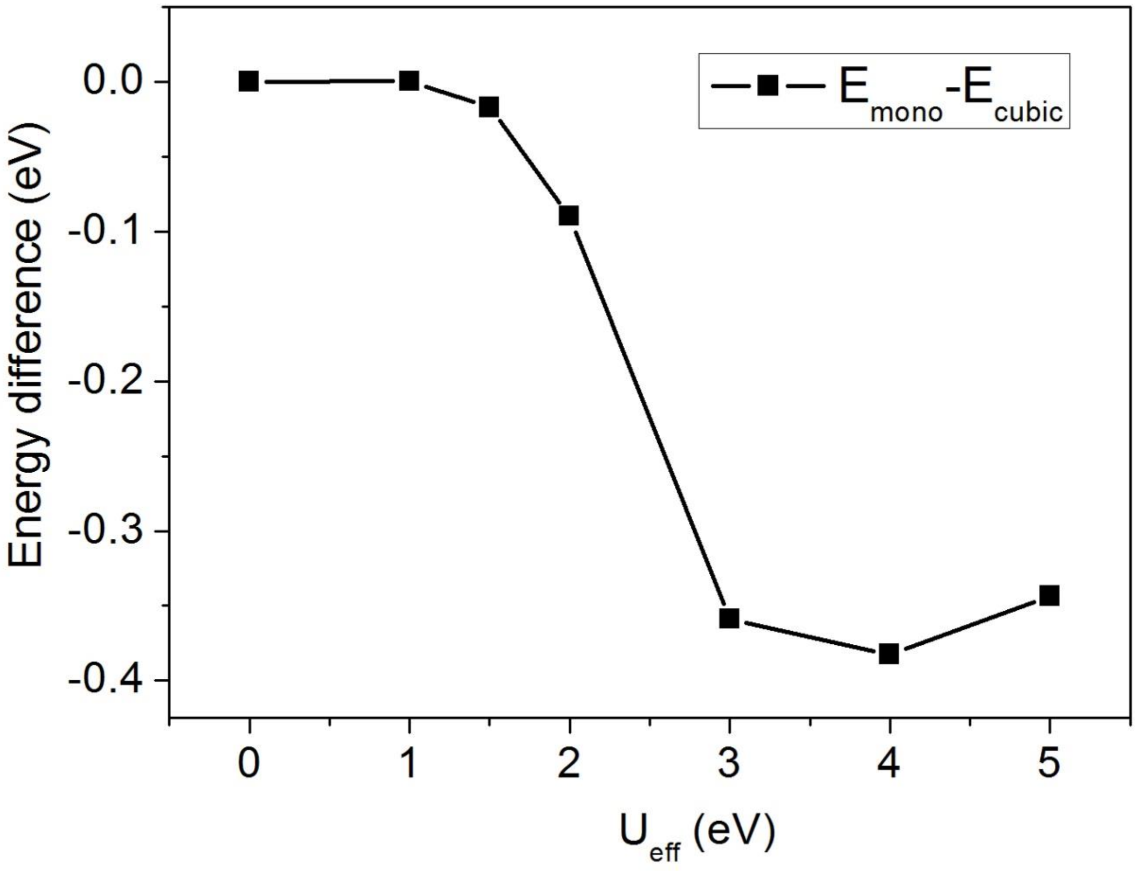

Fig. S5 U_{eff} dependent total energy differences between the monoclinic Fe_3S_4 structures and the cubic Fe_3S_4 structures.
